# Comparison of the Incidence of Postoperative Hypothyroidism in Patients Undergoing Conventional Thyroid Lobectomy and Pyramid- and Isthmus-Preserving Lobectomy

**DOI:** 10.1155/2021/8162307

**Published:** 2021-10-25

**Authors:** Min Jeong Cho, Hyeong Won Yu, Woochul Kim, Yeo Koon Kim, Sang Il Choi, Su-jin Kim, Young Jun Chai, Doohee Lee, Sang Joon Park, June Young Choi, Kyu Eun Lee

**Affiliations:** ^1^Department of Surgery, Seoul National University Bundang Hospital, 82, Gumi-ro 173 Beon-gil, Bundang-gu, Seongnam-si, Gyeonggi-do, Republic of Korea; ^2^Department of Surgery, Seoul National University Bundang Hospital and College of Medicine, 82, Gumi-ro 173 Beon-gil, Bundang-gu, Seongnam-si, Gyeonggi-do, Republic of Korea; ^3^Department of Radiology, Seoul National University Bundang Hospital and College of Medicine, 82, Gumi-ro 173 Beon-gil, Bundang-gu, Seongnam-si, Gyeonggi-do, Republic of Korea; ^4^Department of Surgery, Seoul National University Hospital and College of Medicine, 101 Daehak-ro, Jongno-gu, Seoul, Republic of Korea; ^5^Department of Surgery, Seoul National University Boramae Medical Center and College of Medicine, 20 Boramae-ro 5-gil, Dongjak-gu, Seoul, Republic of Korea; ^6^Department of Research and Development, MEDICALIP Co. Ltd., Seoul, Republic of Korea; ^7^Department of Radiology, Seoul National University College of Medicine, 101 Daehak-ro, Jongno-gu, Seoul, Republic of Korea

## Abstract

Hypothyroidism is a recognized sequela of conventional thyroid lobectomy. However, there have been no studies on the incidence of hypothyroidism following the preservation of the isthmus and pyramid during lobectomy. Therefore, in the present study, we compared the incidence of hypothyroidism following conventional lobectomy and lobectomy during which the isthmus and pyramidal lobe were preserved. Data for a total of 65 patients collected between September 2018 and April 2019 were reviewed retrospectively. Circulating thyroid-stimulating hormone (TSH) concentration was measured before and after surgery in a group who underwent conventional thyroid lobectomy (*n* = 29) and in a group in which the isthmus and pyramid were preserved (*n* = 36). We found no significant difference in TSH concentration between the two groups before surgery, or 3 months or 1 year after surgery. Thus, there might be no difference in the incidence of postoperative hypothyroidism between patients who undergo conventional thyroid lobectomy and those in which the isthmus and pyramid are preserved.

## 1. Introduction

Thyroid nodules, which include benign and malignant thyroid tumors, are treated surgically [1, 2]. The conventional surgical method of thyroid lobectomy involves the removal of approximately half of the thyroid gland by resection along the center of the isthmus [3]. The pyramidal lobe is usually located in the left thyroid gland and may or may not be excised during thyroid lobectomy, according to the surgeon's preference. According to the published literature, 22–85% of patients who undergo thyroid lobectomy develop hypothyroidism, for which the thyroid hormone is required for the restoration and maintenance of euthyroid status [4, 5]. In several previous studies, postoperative hypothyroidism has been documented following thyroid lobectomy. Furthermore, the volume of the residual thyroid and preoperative thyroid-stimulating hormone (TSH) concentration have been shown to be associated with the development of postoperative hypothyroidism [6, 7].

These findings led to speculation that the incidence of postoperative hypothyroidism would be reduced if a sufficiently large volume of the thyroid gland is preserved. In a previous study, the surgeons aimed to increase the residual thyroid volume by preserving the entire isthmus during conventional thyroid lobectomy [8]. The authors concluded that postoperative hypothyroidism following conventional thyroid lobectomy appears to be very rare when the isthmus is preserved. However, in this study, there was only a single patient group, in which the left isthmus was preserved, and thus, there was no comparison with patients undergoing conventional thyroid lobectomy under similar circumstances. Furthermore, the pyramidal lobe was not preserved. Thus, to date, there have been no studies on the effects of preserving both the isthmus and pyramid on the incidence of postoperative hypothyroidism.

In the present study, we aimed to utilize a surgical method that leaves both the isthmus and pyramidal lobe intact when performing thyroid lobectomy and to compare the incidence of hypothyroidism in patients who underwent this procedure with those who underwent conventional thyroid lobectomy.

## 2. Materials and Methods

### 2.1. Study Design

We performed a retrospective review of data relating to patients who underwent thyroid lobectomy at Seoul National University Bundang Hospital between September 2018 and April 2019. All surgeries were performed by two expert endocrine surgeons. The study was approved by the institutional ethics committee (approval number: B-2101/661-102), but the requirement for informed consent was waived, owing to the retrospective nature of the study.

**Figure 1:**
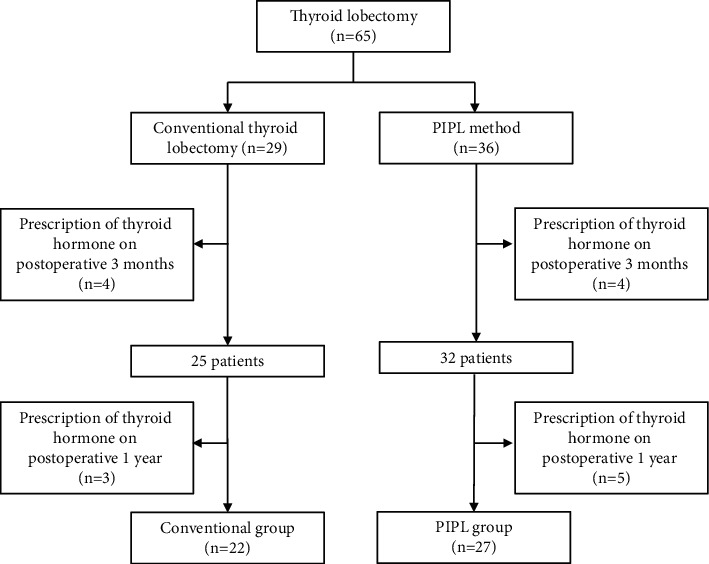
Study design. PIPL, pyramid- and isthmus-preserving lobectomy.

All but one of the participants had thyroid cancer, and the last had a benign thyroid nodule. All the thyroid lobectomy procedures were performed when a sufficient margin was secured from the thyroid capsule. The participants were allocated to two groups on the basis of having undergone conventional thyroid lobectomy or surgery that involved pyramid- and isthmus-preserving lobectomy (PIPL) (Figure 1). Because it is already known that leaving some of the isthmus and pyramid lobe does not affect the recurrence of thyroid cancer, PIPL surgery was performed only when there was no nodule in the isthmus and a sufficient resection margin of 2 mm or more was secured.

The patient's sex, age, circulating TSH concentration, computed tomography (CT) scan data, and details of the prescribed drugs were recorded. All patients in Korea undergo a CT scan with a contrast medium (Omnipaque, GE Healthcare, United States) before thyroid surgery. The CT scan provides anatomical information on features such as arteriosus lusoria and can detect lymph node metastasis. TSH was measured before surgery, and 3 months and 1 year after surgery. Three-dimensional reconstruction of the thyroid gland was performed using the preoperative thyroid CT data. Using this method, the volume of the resected and remaining portions of the thyroid gland was measured. None of the participants took levothyroxine before surgery, and those who were taking this drug 3 months or 1 year after the surgery were excluded from the study. A total of eight patients started thyroid hormone therapy 3 months after surgery. Among these, four started therapy because of an increase in the circulating TSH concentration and four because they requested it based on a family history of thyroid cancer or were preparing for pregnancy. An additional eight patients started thyroid hormone therapy 1 year after surgery due to elevated TSH levels.

### 2.2. Difference between Conventional Lobectomy and PIPL Lobectomy

**Figure 2:**
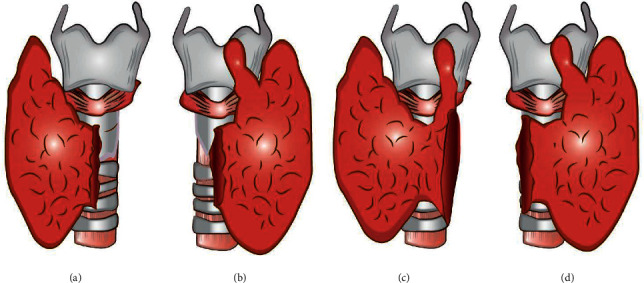
Illustrations of the preserved portions of the thyroid gland following conventional thyroid lobectomy and pyramid- and isthmus-preserving lobectomy (PIPL). (a) Left conventional thyroid lobectomy. (b) Right conventional thyroid lobectomy. (c) Left PIPL. (d) Right PIPL.

We performed conventional thyroid lobectomy, during which the central part of the thyroid gland and ∼50% of the total volume was resected, and PIPL lobectomy, during which the isthmus and pyramid lobes are preserved, such that >50% of the total volume remains. PIPL lobectomy was performed for both the benign thyroid nodule and for small, intrathyroidal papillary thyroid cancer (Figure 2).

### 2.3. Thyroid Volume Measurement

**Figure 3:**
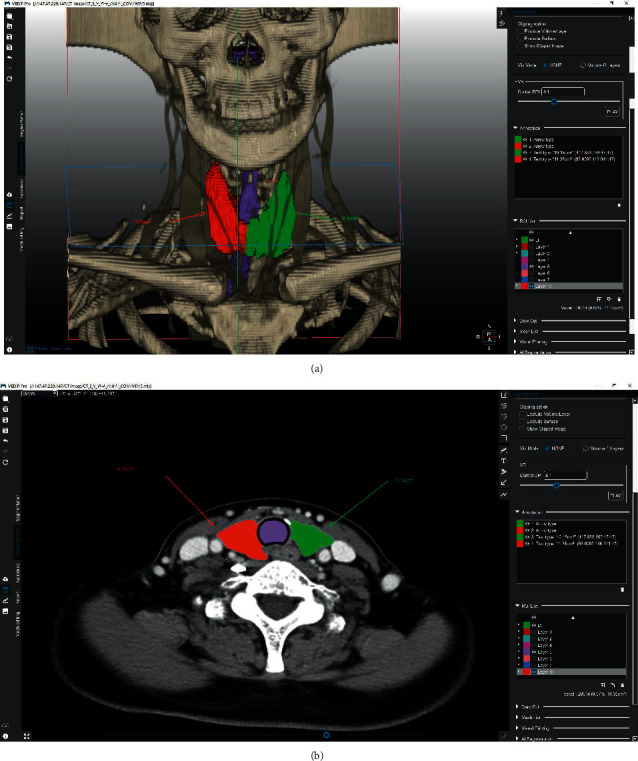
Representative image of the volumetric extraction of the thyroid. (a) 3D image shows a volumetric segmentation of the right (green) and left (red) thyroid glands using a Draw-Cut function. (b) Axial image shows the results of segmentation. The 3D segmentation volumes were measured using MEDIP software.

To measure the volume of the thyroid portions, the thyroid was initially segmented using an automated process that involved machine learning-based threshold and graph-cut algorithms (MEDIP®, http://medicalip.com/Medip, MEDICALIP, Seoul, Republic of Korea). CT DICOM images of the patients were registered in MEDIP, and preprocessing was initially used to remove the noise associated with the diagnostic image and improve image quality. Subsequently, the user designated a region of interest (ROI), which was replaced with seed information, and then, segmentation was performed for this area using the graph-cut algorithm, which separates the foreground from the background by configuring each pixel with graph intersections (nodes), utilizing the difference in energy (flow network) between them. Segmentation was performed using a seed point specified by the user as the starting point. The foreground and background were specified by the user by drawing a solid line with a pencil via an input device such as a mouse (Sketch method), creating a polygon by clicking on the coordinates and filling it inside (Polygon method), or filling the inside in free-form manner (Freedraw method [9]. Semiautomated segmentation was performed using the Draw-Cut function in MEDIP and used to reconstruct the thyroid in three dimensions (Figure 3). After classifying the volume of the thyroid gland, a virtual line was drawn in three dimensions using the software at the margin of the resected portion of the gland. Afterwards, the resected and remaining portions of the thyroid gland were displayed and the volume of each was measured using the automatic volume measurement function. The thyroid segmentation and visual printing (VP) process using MEDIP® software are shown in Video S1.

### 2.4. Definition of Hypothyroidism

Hypothyroidism was defined as a TSH concentration greater than 4.1, and subclinical hypothyroidism was defined as a TSH concentration between 4.1 and 8 without clinical symptoms.

### 2.5. Statistical Analysis

Chi-square testing was performed for dichotomous variables, and Student's *t*-test was performed for continuous variables to compare the conventional group with the PIPL group. All statistical analyses were performed using SPSS version 18.0 (IBM, Inc., Armonk, NY, USA). *p* < 0.05 was considered to represent statistical significance.

## 3. Results

Summary data for the participants are presented in Table 1. A total of 65 participants underwent thyroid lobectomy, of which 29 were in the conventional group and 36 in the PIPL group. In the conventional group, there were 23 women (79.3%), and in the PIPL group, 25 women (69.4%). The mean age of the conventional group was 40 years, and that of the PIPL group was 39 years. The number of participants who underwent right thyroid lobectomy was 19 (66%) and 18 (50%) in the conventional and PIPL groups, respectively, and the number who underwent left thyroid lobectomy was 10 (34%) and 18 (50%), respectively. The mean total volume was 19,784 mm^3^ for the conventional group and 16,623 mm^3^ for the PIPL group. The mean resected volume was 10,241 mm^3^ for the conventional group (52% of the preoperative volume) and 7,651 mm^3^ for the PIPL group (46% of the preoperative volume). The residual volume was 9,543 mm^3^ for the conventional group (48% of the preoperative volume) and 8,972 mm^3^ for the PIPL group (54% of the preoperative volume).

**Figure 4:**
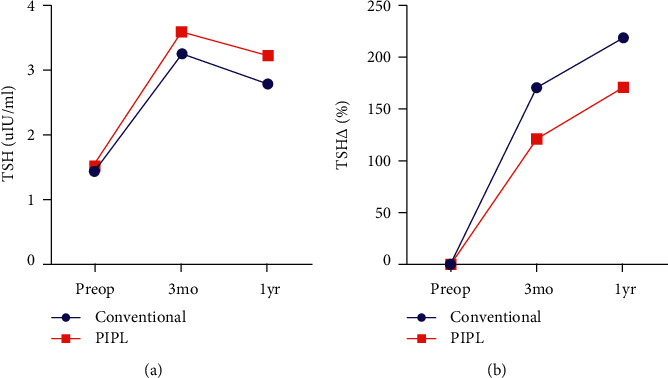
Mean thyroid-stimulating hormone (TSH) concentrations and changes during the study in the conventional and PIPL groups. (a) Serum TSH concentration before surgery, and 3 months and 1 year following thyroid surgery. (b) Changes in the serum TSH concentration during the first year following thyroid surgery.

Table 2 and Figure 4(a) show the TSH concentrations before and after thyroid surgery and the differences between these values. The mean TSH concentration of the conventional group before surgery was 1.45 *μ*IU/ml, and that of the PIPL group was 1.52 *μ*IU/ml. Three months after surgery, the mean TSH concentration of the conventional group was 3.25 *μ*IU/ml and that of the PIPL group was 3.4 *μ*IU/ml (*p*=0.701). One year after surgery, the mean TSH concentration of the conventional group was 3.41 *μ*IU/ml and that of the PIPL group was 3.74 *μ*IU/ml (*p*=0.451).

The mean free T4 concentration of the conventional group before surgery was 1.19 ng/dL, and that of the PIPL group was 1.25 ng/dL. Three months after surgery, the mean free T4 concentration of the conventional group was 1.16 ng/dL and that of the PIPL group was 1.2 ng/dL (*p*=0.295). One year after surgery, the mean free T4 concentration of the conventional group was 1.19 ng/dL and that of the PIPL group was 1.16 ng/dL (*p*=0.075).

The mean total T3 concentration of the conventional group before surgery was 134 *μ*g/dL and that of the PIPL group was 142 *μ*g/dL. Three months after surgery, the mean total T3 concentration of the conventional group was 105 *μ*g/dL and that of the PIPL group was 113 *μ*g/dL (*p*=0.478). One year after surgery, the mean total T3 concentration of the conventional group was 120 *μ*g/dL and that of the PIPL group was 91 *μ*g/dL (*p* < 0.001).

The mean changes in TSH concentration up to 3 months following surgery were 1.9 *μ*IU/ml in the conventional group and 1.7 *μ*IU/ml in the PIPL group (*p*=0.518). The mean changes up to 1 year following surgery were 2.0 *μ*IU/ml for the conventional group and 2.3 *μ*IU/ml for the PIPL group (*p*=0.891).

The mean percentage changes in TSH concentration up to 3 months following surgery were 1.71% for the conventional group and 1.22% for the PIPL group (*p*=0.069). The mean percentage changes in TSH concentration up to 1 year were 2.19% for the conventional group and 1.71% for the PIPL group (*p*=0.271). Thus, the conventional group tended to show larger increases than the PIPL group (Figure 4(b)).

TSH concentrations of 0.4–4.1 *μ*IU/ml were defined as normal, and hypothyroidism was diagnosed when the concentration was >4.1 *μ*IU/m.  Table 3 shows the incidences of hypothyroidism before and after thyroid surgery in each group. Chi-square testing was used to analyze the data, and this showed no significant differences between the groups (*p*=0.298, 3 months after thyroid surgery and *p*=0.257, 1 year after thyroid surgery).

## 4. Discussion

The thyroid gland is an endocrine organ that secretes thyroid hormones, which have a range of essential functions [10]. For some benign thyroid nodules, lobectomy is performed for diagnostic or therapeutic purposes. In addition, lobectomy is performed for the treatment of well-differentiated thyroid cancer nodules of <4 cm in size [11]. Theoretically, subsequent levothyroxine therapy should not be required, because half of the total thyroid volume is preserved during thyroidectomy. However, in reality, 6.5–45% of patients develop hypothyroidism and require levothyroxine therapy [8]. There have been many studies on the hypothyroidism that develops following thyroid lobectomy, and it is thought to arise because of the inadequate remaining thyroid volume [6] or preoperative TSH concentration [5]. Therefore, it has been hypothesized that the preservation of thyroid volume would be associated with superior thyroid function, but no objective comparisons of surgical techniques have been performed to date [6]. In the present retrospective study, we compared patients who underwent conventional thyroid lobectomy, which involved the removal of ∼50% of the existing thyroid volume, and a new surgical method that involves the retention of a larger thyroid volume.

According to previous anatomical studies, an isthmus is present in the thyroid gland in 92.5% of adults and it is 11.1 ± 6.2 cm × 15.9 ± 5.8 cm in size [8, 9]. In addition, a pyramid is present in 38.7–52.5% of adults and is approximately 22.6 cm × 11.2 cm in size [11–15]. Furthermore, in 60–65% of people, the pyramid is on the left [16]. During conventional thyroid lobectomy, half of the isthmus is excised and the pyramid may be either excised or preserved. In comparison to the right and left thyroid glands, the isthmus and pyramid have small but significant volumes. According to previous reports, the remaining pyramid lobe is sufficient to affect the direction of patient follow-up after surgery [17]. Therefore, we have used a surgical method that preserves the pyramid and isthmus and should be associated with the retention of a larger volume of the gland as a whole. We hypothesized that the incidence of postoperative hypothyroidism would be lower if a larger volume of the thyroid was preserved.

We analyzed the thyroid CT images of patients who had undergone conventional thyroid lobectomy and others who had undergone PIPL. Their thyroid glands were reconstructed in three dimensions using MEDIP software, in which an imaginary line was drawn that would preserve the pyramid and isthmus, and then, the volumes of the resected and remaining portions of the thyroid gland were measured and the ratio of the two analyzed. In this way, we showed that in the group that underwent conventional thyroid lobectomy, ∼52% of the thyroid volume was resected, with ∼48% of the volume remaining. In contrast, in the PIPL group, ∼46% of the thyroid volume was excised and ∼54% was preserved. After surgery, none of the participants took levothyroxine, the circulating TSH concentration was remeasured after 3 months and 1 year, and these concentrations were compared with the preoperative concentration. We found no difference in the TSH concentration between the two groups, and there were also no differences in the changes in the TSH concentration following surgery. A TSH concentration >4.1 *μ*IU/ml was used to define hypothyroidism, and no difference in the postoperative incidence of hypothyroidism was identified between the two groups.

In the present study, for the first time, we compared the incidences of hypothyroidism following surgery in patients who underwent either conventional thyroid lobectomy or PIPL lobectomy and found no significant difference. However, it should be mentioned that the study was limited by a relatively small sample size and its retrospective nature. Therefore, in the future, a large-scale, controlled, prospective study should be conducted to corroborate the present findings.

## 5. Conclusions

We have designed a new method of thyroid lobectomy that preserves the isthmus and pyramid. However, the use of this technique might not be associated with a lower incidence of postoperative hypothyroidism than conventional thyroid lobectomy.

## Figures and Tables

**Table 1:** Summary data for the participants.

	Conventional group (29)	PIPL group (36)	*p* value
Sex (male, %)	6 (26%)	11 (44%)	0.368
Age (mean ± SD)	40 ± 2.02	39 ± 1.70	0.775
Surgery			0.209
Right thyroid lobectomy (*n*, %)	19 (66%)	18 (50%)	
Left thyroid lobectomy (*n*, %)	10 (34%)	18 (50%)	
Thyroid volume			
Total volume (mm^3^, %)	19,784 (100%)	16,623 (100%)	0.074
Resected volume (mm^3^, %)	10,241 (52%)	7,651 (46%)	0.02
Remnant volume (mm^3^, %)	9,543 (48%)	8,972 (54%)	0.012
Pathology			
Papillary carcinoma (*n*, %)	27 (93.3%)	35 (97%)	
Follicular carcinoma (*n*, %)	1 (3.4%)	0 (0%)	
Nodular hyperplasia (*n*, %)	1 (3.4%)	1 (3%)	

PIPL, pyramid- and isthmus-preserving lobectomy; SD, standard deviation.

**Table 2:** Comparisons of the mean TSH concentrations before surgery, and 3 months and 1 year after surgery.

	Conventional group	PIPL group	*p* value
Before surgery (*n* = 65)	1.45 (29)	1.52 (36)	0.658
3 months after surgery (*n* = 57)			
TSH_3mo	3.25 (25)	3.4 (32)	0.701
TSH_Δ3mo	1.9 (25)	1.7 (32)	0.518
TSH_Δ%	1.71 (25)	1.22 (32)	0.069
1 year after surgery (*n* = 49)			
TSH_1yr	3.41 (22)	3.74 (27)	0.451
TSH_Δ1yr	2.0 (22)	2.3 (27)	0.891
TSH_Δ%	2.19 (22)	1.71 (27)	0.271

PIPL, pyramid- and isthmus-preserving lobectomy; TSH, thyroid-stimulating hormone; mo, month; yr, year.

**Table 3:** Incidences of hypothyroidism and euthyroidism after thyroid surgery in the conventional and PIPL groups.

		Conventional group	PIPL group	*p* value
3 months after surgery	Hypothyroidism	7 (24.1%)	13 (36.1%)	0.298
	Euthyroidism	22 (75.9%)	23 (63.9%)	

1 year after surgery	Hypothyroidism	6 (20.7%)	12 (33.3%)	0.257
	Euthyroidism	23 (79.3%)	24 (66.6%)	

PIPL, pyramid- and isthmus-preserving lobectomy.

## Data Availability

This study is a retrospective study, and data were not permitted to be disclosed.

## Supplementary Materials

Supplementary Materials Video S1: video of thyroid segmentation and visual printing (VP) on CT DICOM images of patients using MEDIP® software. The video on the left is that of the conventional group, and the one on the right is that of the pyramid- and isthmus-preserving lobectomy group.
