# Supplementary material for: Comparison of the Incidence of Postoperative Hypothyroidism in Patients Undergoing Conventional Thyroid Lobectomy and Pyramid- and Isthmus-Preserving Lobectomy
Source: Int J Endocrinol. 2021 Oct 25;2021:8162307. doi: 10.1155/2021/8162307 (PMC8560291; doi:10.1155/2021/8162307)
Supplement: Supplementary Materials — Video S1: video of thyroid segmentation and visual printing (VP) on CT DICOM images of patients using MEDIP® software. The video on the left is that of the conventional group, and the one on the right is that of the pyramid- and isthmus-preserving lobectomy group. [file 8162307.f1.docx]

https://1drv.ms/v/s!AlcrfHt8B9EqnHRuph2fkUWODLxd?e=VgZ58N
